# Itraconazole as a repurposed anti-cancer agent: focusing on synthesis, mechanisms of action and therapeutic insights

**DOI:** 10.1039/d5ra09799a

**Published:** 2026-05-14

**Authors:** Mahrokh Marzi, Abdolmajid Ghasemian, Ali Ghanbariasad, Ensieh Nournia, Elham Zarenezhad

**Affiliations:** a Noncommunicable Diseases Research Center, Fasa University of Medical Sciences Fasa Iran El.zarenezhad@gmail.com +98 715 331 6324; b Department of Medical Biotechnology, School of Medicine, Fasa University of Medical Sciences Fasa Iran

## Abstract

Cancer drug development faces significant challenges due to long timelines, stringent regulatory requirements, and high failure rates. Drug repurposing has emerged as an efficient strategy to accelerate the discovery of novel therapies. Itraconazole (ITZ), a triazole antifungal approved in 1992 and widely used for over 30 years, has recently shown promising anticancer activity. This review provides a comprehensive overview of ITZ in cancer therapy, covering its synthesis and chemistry-based perspectives, mechanisms of action including angiogenesis inhibition and modulation of key signaling pathways, and preclinical and clinical evidence of its efficacy against various cancers. In addition, we discuss comparative drug delivery systems to optimize its therapeutic potential and summarize the latest studies from 2021 to 2025 highlighting its role as a potential anticancer agent. By consolidating current knowledge, this review aims to guide researchers and clinicians in exploring ITZ as a repurposed therapeutic option for cancer treatment.

## Abstract 2

Cancer drug development faces significant challenges due to long timelines, stringent regulatory requirements, and high failure rates.

## Graphical abstract

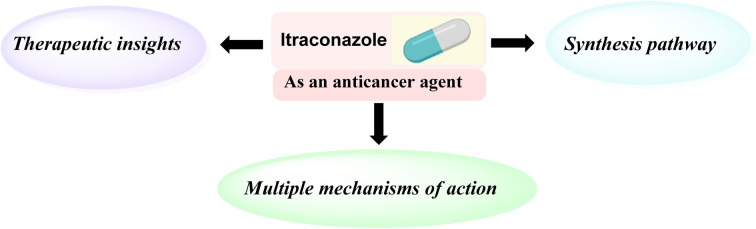

## Introduction

In 2020, approximately 19.3 million new cases of cancer were reported worldwide, resulting in nearly 10 million fatalities due to the illness.^1^ Cancer remains a significant public health challenge in the United States. The American Cancer Society (ACS) projects approximately 2.04 million new cancer diagnoses and 618 120 cancer-related deaths in 2025. This increase in cases compared to earlier years reflects changing population dynamics and the ongoing presence of risk factors.^2^ Despite advancements, cancer prevention efforts are falling behind. Incidence rates continue to increase for six of the ten most common cancers—breast, prostate, melanoma, uterine corpus, pancreatic, and colorectal cancer (among those under 65)—with two of these cancers primarily affecting women.^2^ Enhanced cancer control measures and investment in better early detection and treatment will help decrease cancer mortality rates. Although significant strides have been achieved in cancer treatment, it continues to be a serious issue worldwide and requires innovative therapies.^3^ The improvement of anticancer drugs or novel drug development need long time and costs. After distinguishing or synthesizing a modern compound, it must pass through preclinical testing and numerous stages of clinical trials (stage I, II, and III) before it can be approved. Drug repurposing, on the other hand, focuses on finding new medical applications for drugs that are already available.^4^ Since the pharmacokinetics, pharmacodynamics, and safety of a drug in humans are already established, repurposing it for different diseases can save both time and costs.

Examining the anticancer impacts and fundamental components of FDA-approved drugs not initially planned for cancer, such as their capacity to balance irregular cell signaling pathways and boost the antitumor safe response, offers a promising procedure for quickening and lessening the taken a toll of anticancer sedate advancement. This approach seems to offer assistance in overcoming the challenges confronted by customary cancer drug research and development.^5^

Azoles are five-membered heterocyclic compounds that include nitrogen atoms and rank among the most commercially successful in their category. The presence of two nitrogen atoms in the azole ring imparts distinctive characteristics that significantly influence their structural diversity and biological activities.^6^ Triazoles act as bioisosteres for amides, esters, and carboxylic acids as they weakly interact with proteins, enzymes, and receptors in biological systems.^7^

ITZ (C_35_H_38_C_l2_N_8_O_4_), a broad-spectrum antifungal drug approved by the FDA and belonging to the triazole class, has been used clinically for over 30 years. While screening a collection of compounds known to inhibit human endothelial cell proliferation to find effective angiogenesis inhibitors, Chong and colleagues unexpectedly identified ITZ as a promising anti-angiogenic agent.^8^ It functions by blocking the activity of lanosterol 14α-demethylase (CYP51), a cytochrome P450-dependent enzyme that plays a key role in the biosynthesis of ergosterol in fungi.^6^ However, ITZ's antifungal activity is likely independent of its anticancer effects.^9^ Evidence from both preclinical and clinical research supports its potential as an anticancer agent, either used on its own or in combination with other chemotherapeutic drugs.^9^ This review emphasizes synthesis of ITZ, it's role in cancer treatment, mechanism of action and provides an overview of ongoing clinical trials. This study may be valuable to professionals such as chemists, pharmacists, pharmacologists, and medicinal chemists.

## Methods

**Fig. 1:**
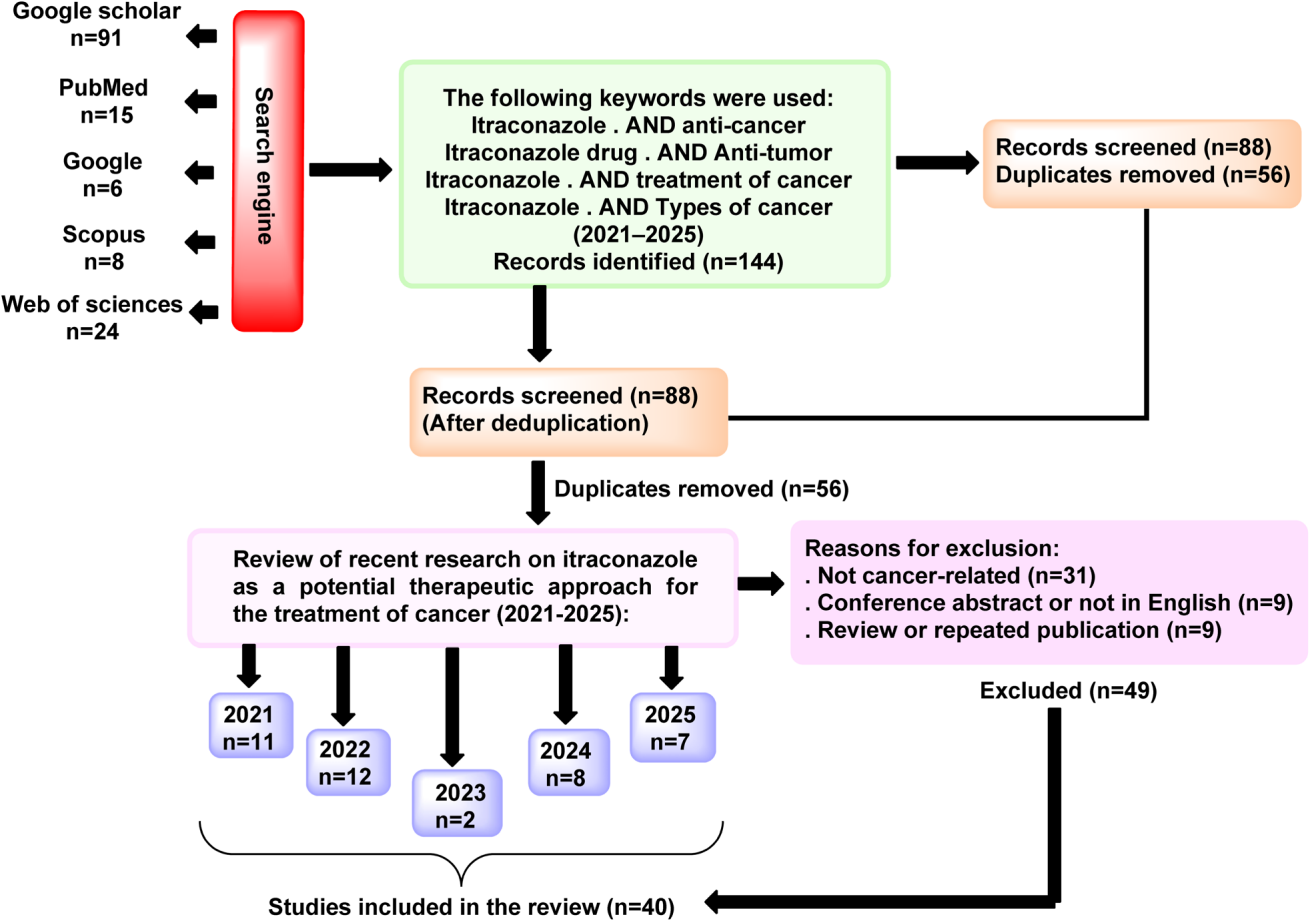
Content charts provided in this review.

This research focuses on the latest findings (2021–2025) about ITZ as a possible cancer therapy strategy. Details of the selection process for the papers in this study are shown in the figure below (Fig. 1). The literature search is conducted using the Web of Science, Scopus, Google, PubMed, and Google Scholar databases. The keywords listed below were used: itraconazole * anti-cancer, itraconazole drug * anti-tumor, itraconazole * treatment of cancer, itraconazole * types of cancer (2021–2025).

## Application of itraconazole

Triazole antifungal drug with a broad spectrum ITZ comes in oral and intravenous formulations and has favorable pharmacological and pharmacokinetic characteristics. Over the past 20 years, both human and animal studies have shown how well it works to treat a range of superficial fungal infections, including challenging cases of dermatophytosis and onychomycoses.^10^ Additionally, shorter treatment plans have demonstrated its potential. For instance, one-week pulse therapy, which is given once a month for two to four months to treat onychomycosis and follicular dermatophytosis, has been demonstrated to be effective in treating vaginal candidiasis. Despite the paucity of clinical data about deep mycoses, ITZ exhibits promise in the treatment of systemic candidiasis, sporotrichosis, blastomycosis, paracoccidioidomycosis, aspergillosis, and certain cases of histoplasmosis. ITZ is still useful in treating chromomycosis and coccidioidomycosis, despite its decreased efficacy against these refractory conditions. When taking ITZ with other medications, caution should be exercised because there may be drug interactions mediated by the cytochrome P450 enzyme 3A4 system, even though dosages up to 400 mg day^−1^ are usually well-tolerated and rarely result in significant adverse effects.^11^

## Synthesis of itraconazole

According to Heeres *et al.*'s method^12,13^ for synthesizing ITZ, compound 3 was produced by reacting 2,4-dichloro acetophenone 1 with glycerol 2, then brominating the result to produce 4. Benzoylation of compound 4 produced compound 5, which was further hydrolyzed and treated with 1,2,4-triazole to provide product 6. Compound 6, which is necessary for the production of ITZ, was mesitylated to create racemic building block 7. After piperidine 8 was alkylated with *p*-chloronitrobenzene 9 and reduced by catalytic hydrogenation, compound 11 was produced. The cyclization of compound 13 with formamidine produced compound 14, which was further alkylated using racemic 2-bromobutane and HBr demethylation to produce compound 16, which combined with compound 7 to produce ITZ 17.^14^

**Scheme 1:**
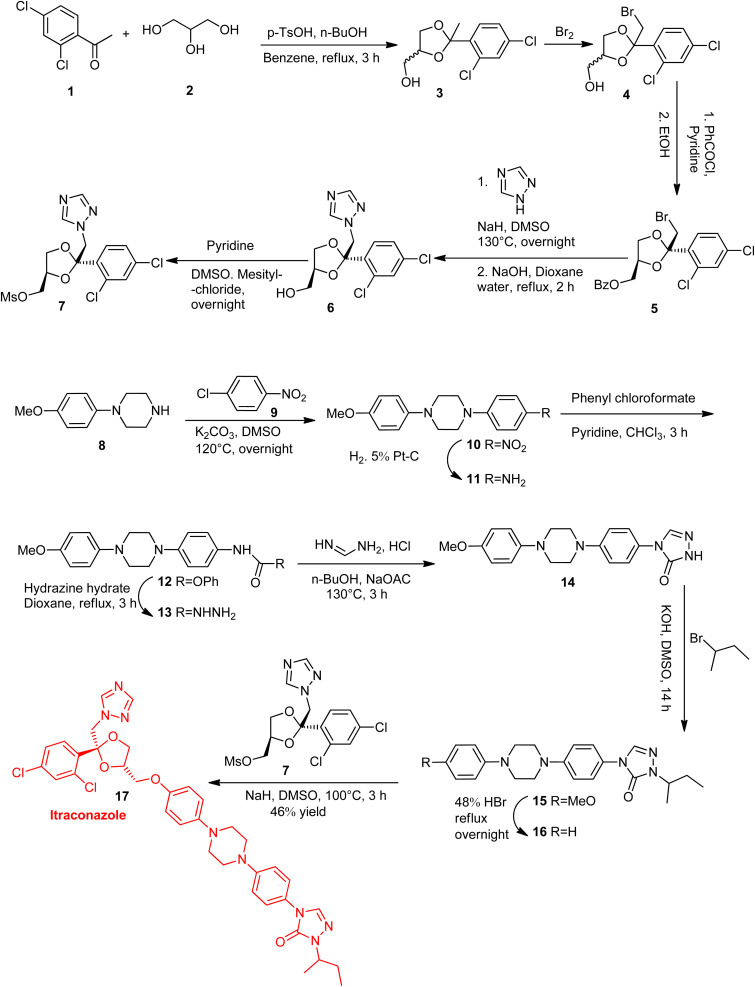
[Itraconazole synthesis], reproduced from ref. 14 with permission from Elsevier, *Tetrahedron*, and ref. 13 with permission from Elsevier, *Journal of Medicinal Chemistry*, copyright 2026.

In addition to the classical multi-step synthesis described by Heeres *et al.*,^12,13^ recent research in ITZ manufacturing has focused on process innovations that improve scalability and product quality in pharmaceutical production. For example, bottom-up anti-solvent crystallization combined with membrane diafiltration has been developed to enhance downstream processing of ITZ crystal suspensions by significantly reducing residual solvent levels and optimizing excipient content without compromising crystal stability, offering a scalable approach for long-acting injectable formulations. Moreover, continuous processing strategies such as hot-melt extrusion (HME) and spray drying have been applied to produce amorphous solid dispersions (ASDs) of ITZ with improved dissolution and bioavailability. These continuous manufacturing techniques, particularly HME, are increasingly used in industry due to solvent-free operation, enhanced content uniformity, and scalability for oral solid dosage forms. Furthermore, twin-screw extrusion with mesoporous carriers has been shown to achieve substantial amorphization of ITZ under solvent-free conditions, demonstrating emerging continuous approaches for improving API properties (Scheme 1).^15–17^

## Chemistry-based perspectives on itraconazole repurposing

The multifunctional anticancer action of ITZ is made possible by its structurally complex and extremely lipophilic scaffold, which includes a triazole ring, dioxolane moiety, several aromatic groups, and a flexible side chain. These characteristics enable interactions with a variety of biological targets, giving ITZ a chemical foundation for controlling angiogenesis, autophagy, cholesterol transport, and Hedgehog (Hh) signaling.^18^

Reducing CYP3A4 inhibition while maintaining anticancer activity presents optimization opportunities from a medicinal chemistry perspective. Promising methods to increase safety and selectivity include side-chain simplification, structural alteration of the triazole motif, and modulation of aromatic substitutions. The production of anticancer-oriented analogs with reduced antifungal activity is made possible by the proven synthetic pathway of ITZ, which permits late-stage diversification.

According to preliminary structure–activity relationship (SAR) research, hydrophobic bulk and steric characteristics—rather than only the traditional azole pharmacophore—are the main drivers of anticancer effects. These results underline the significance of chemistry-guided optimization in developing ITZ as a repurposed anticancer drug and suggest the viability of creating ITZ-derived or non-azole analogs with enhanced therapeutic characteristics.^18,19^

## Mechanisms of action

Various anticancer effects of ITZ have been explained by a number of different mechanisms of action. These consist of: (1) autophagy induction, (2) anti-angiogenesis, (3) reversal of multi-drug resistance, and (4) Hh pathway inhibition.^20^

**Fig. 2:**
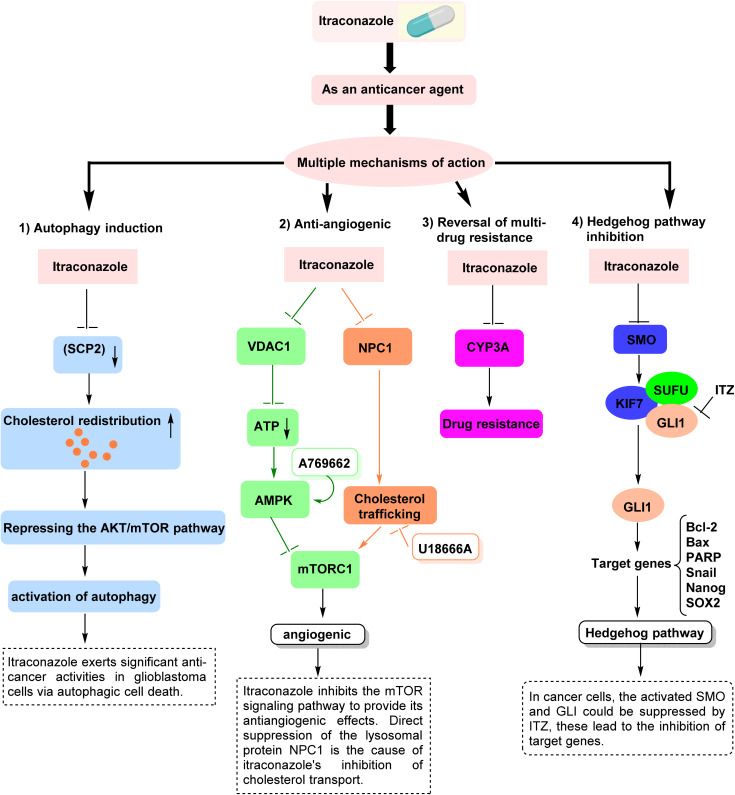
[Anticancer mechanisms of ITZ. (1) Autophagy induction: ITZ suppresses the AKT/mTOR pathway, promoting autophagic cell death and inhibiting glioblastoma cell growth, partly through altered cholesterol trafficking. (2) Anti-angiogenic activity: ITZ inhibits angiogenesis *via* AMPK activation and mTOR suppression, mediated by VDAC1 targeting and disruption of cholesterol transport through NPC1. (3) Reversal of multidrug resistance: ITZ inhibits CYP3A4 activity, contributing to reduced drug metabolism and enhanced chemosensitivity. (4) Hh pathway inhibition: ITZ blocks SMO and GLI signaling, leading to downregulation of oncogenic targets involved in proliferation, survival, angiogenesis, and drug resistance], reproduced from ref. 27 with permission from Elsevier, *ACS Chemical Biology*, and ref. 23 with permission from Elsevier, *Journal of Advanced Research*, copyright 2026.

(1) Autophagy induction: ITZ was discovered to cause autophagic cell growth arrest in C6 and U87 glioblastoma cells in a mouse xenograft (U87) model as well as *in vitro*.^21^ As shown in endothelial cells, the impact is linked to the inhibition of mechanistic target of rapamycin (mTOR) signaling, which is brought on by ITZ's blocking of cholesterol trafficking.^22^ Additionally, AKT1, an upstream regulator of mTOR, was inhibited by ITZ; reactivating AKT1 reversed the induction of growth arrest and autophagy. The decrease in cellular proliferation was reversed by autophagy inhibition, indicating that using ITZ in conjunction with autophagy inhibitors may present challenges.^23^

(2) Anti-angiogenic: an investigation into the molecular mechanism of ITZ's antiangiogenic action found that ITZ indirectly suppresses mTOR signaling *via* the 5′ AMP-dependent protein kinase (AMPK), another upstream regulatory kinase.^24^ A decrease in cellular energy levels was demonstrated to be the consequence of ITZ direct binding to and blockage of the mitochondrial Voltage-Dependent Anion Channel 1 (VDAC1), a crucial regulator of mitochondrial metabolism, which activates AMPK.^25^ Consequently, it was demonstrated that VDAC1 is a unique target for endothelial cell AMPK/mTOR pathway regulation. In a different study, ITZ was also demonstrated to prevent endothelial cells from transporting cholesterol, which resulted in the buildup of cholesterol in the late endosome/lysosome.^22^ The cells of patients with Niemann–Pick Type C (NPC), a genetic disease in which a lack of one of two lysosomal cholesterol-binding proteins (NPC1 or NPC2) hinder the cholesterol release/leakage from the lysosome to cell cytoplasm (known as the NPC phenotype), and exhibit cholesterol localization defect.^26^ Since mTOR signaling and proliferation in HUVEC were also inhibited by either genetically knocking down NPC1 or NPC2 or by pharmacologically inhibiting cholesterol trafficking with two other known NPC-inducing small molecules, imipramine and U18666A albeit at far higher concentrations than ITZ, it was also deciphered to cease cholesterol trafficking resulting in mTOR inhibition.^26,27^

(3) Reversal of multi-drug resistance: interestingly, there is some substrate overlap for cytochrome P450 monooxygenase (CYP) family member inhibition in azole antifungals. For instance, albeit to varying degrees, posaconazole, ITZ, and ketoconazole all inhibit CYP3A4 activity. The most well-known CYP member implicated in chemotherapy resistance in tumors may be CYP3A. The detoxification of several major anticancer medications in the clinic, such as docetaxel, irinotecan, gefitinib, cisplatin, paclitaxel, tamoxifen, and vinorelbine, is carried out by CYP3A4.^28,29^ Because CYP3A4 overexpression restricts the chemotherapeutic response, CYP3A4 expression downregulation may enhance the therapeutic response.^30^ The suppression of human hepatocyte CYP3A4, the primary cytochrome P450 in the human liver, is a significant drawback of ITZ as a new anticancer drug. About 50% of prescribed medications, including the majority of anticancer medications, are metabolized by the major xenobiotic metabolizing enzyme CYP3A4, which also has important pharmacological and toxicological effects. Changes in catalytic activity are crucial for bioavailability and drug–drug interactions.^31^ Tyrosine kinase inhibitors, which are mostly metabolized by cytochrome P450, are among the majority of anticancer medications whose metabolism is blocked by CYP3A4 inhibition.^32^ Consequently, a number of adverse effects that may result from the suppression of CYP3A4 in the liver should be taken into account when ITZ is used in conjunction with other anticancer medications. Additionally, new ITZ analogs that maintain their antiangiogenic properties with or without CYP3A4 suppression must be developed.^33^

(4) Hedgehog pathway inhibition: in many systems, cancer can result from unchecked Hh pathway activation, which can also cause Hh ligands, smoothened receptors (SMO), and glioma-associated oncogene homologs (GLI) to overexpress and become activated.^34,35^ SMO is essential to the Hh pathway, which controls animal adult stem cells and embryonic development. By blocking SMO and/or GLI, particularly GLI1, and their downstream targets through a variety of mechanisms, ITZ has been shown to suppress the Hh pathway. This has been shown to stop the growth and proliferation of numerous cancers both *in vivo* and *in vitro*, stop the cell cycle, prevent angiogenesis, and trigger apoptosis and autophagy, including in gastric,^36^ liver,^37^ melanoma,^38^ basal cell carcinoma,^39^ prostate,^40^ and other cancers. Furthermore, a number of preclinical investigations have verified that ITZ can treat cancer by blocking the Hh pathway^41,42^ (Fig. 2).

**Fig. 3:**
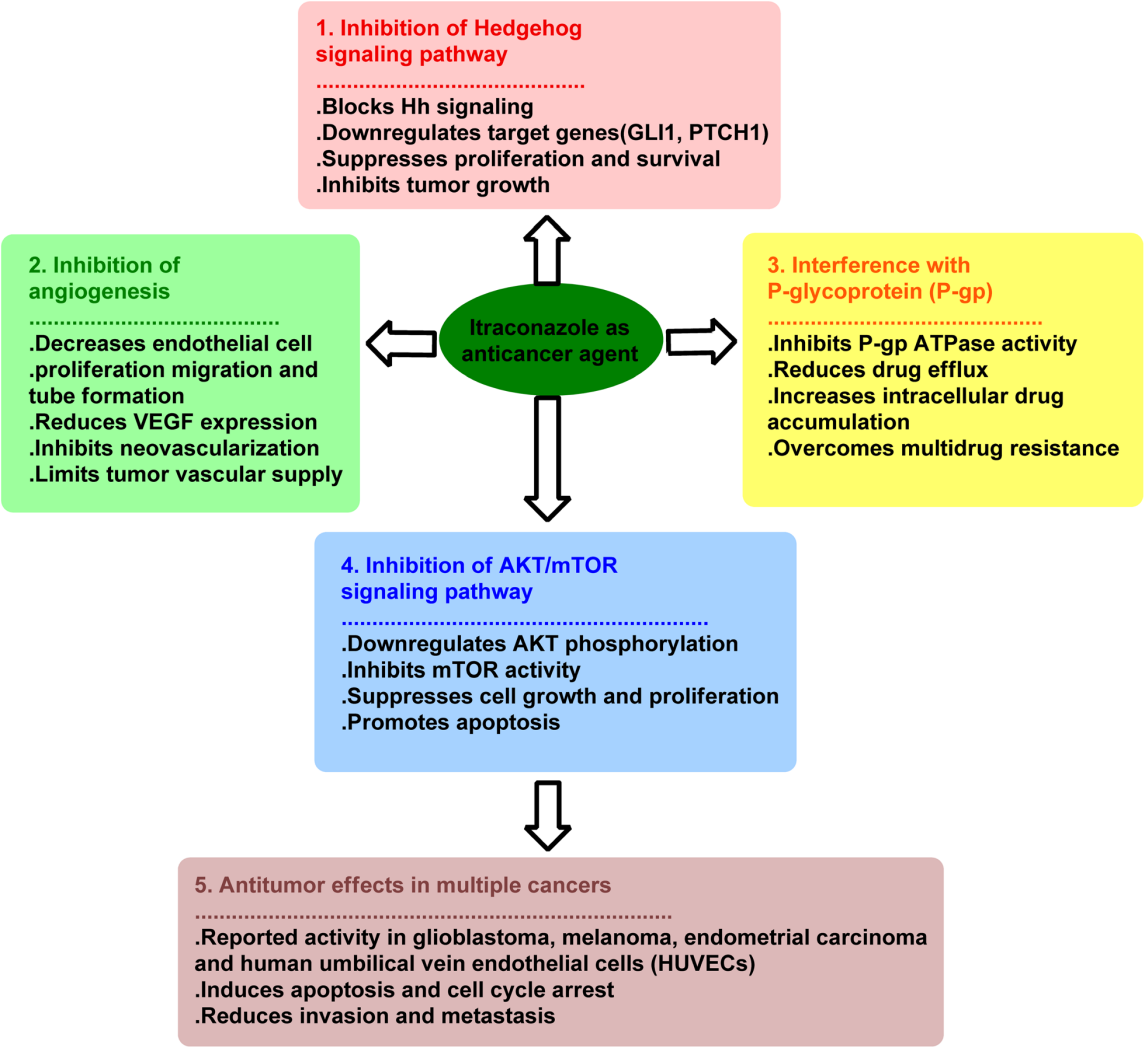
Mechanism of action of Itraconazole in cancer therapy.

A screening analysis for approved small molecule medicines revealed that ITZ inhibited the Hh pathway with an IC_50_ of 690 nM and angiogenesis with an IC_50_ of 160 nM.^41^ Additionally, ITZ suppresses the AKT/mTOR signaling pathway in human umbilical vein endothelial cells (HUVECs), glioblastoma, melanoma cells, and endometrial cancer (EC). It was repurposed as an anticancer medication because it suppresses the chemoresistance caused by P-glycoprotein, regulates Hh signal transduction pathways, and stops cancer cells from angiogenesis. The anticancer mechanism of action of ITZ is depicted in Fig. 3.

## Clinical proof of itraconazole's effectiveness in treating various cancers

Preclinical studies have demonstrated that ITZ possesses anticancer activity across various cancer models. Consequently, several early-phase clinical trials were conducted to evaluate its therapeutic potential in cancer patients. It is becoming more and more appealing to investigate the antitumor effects of current medications. ITZ has been used in clinical settings for many years and is a very safe and well-tolerated antifungal medication. ITZ can cure tumors by blocking Hh signaling, according to earlier research. ITZ's ability to decrease tumors was shown *in vitro* by causing tumor cell growth suppression, autophagy, apoptosis, and cycle arrest. In clinical studies, ITZ has been used as an anti-tumor medication to treat non-small cell lung cancer (NSCLC),^43^ prostate cancer,^40^ and basal cell carcinoma (BCC).^44^ Crucially, ITZ offers unexpected anti-cancer potential to increase patient survival and quality of life in refractory cancers. For instance, combination oral ITZ solution achieved a 57% response rate with 2 full responses and 14 partial responses in patients with metastatic biliary tract cancer following first-line chemotherapy.^45^ Research has indicated that patients with malignant tumors had a higher chance of surviving following ITZ medication. Better curative results can be obtained when ITZ is coupled with other chemotherapy medications (Table 1).^46^

**Table 1:** Examples of preclinical and clinical trials of itraconazole for the treatment of malignant^a^

Cancer type	Study title	Identifier	Phase	Interventions	Primary outcome measures	Status
Recurrent non-small cell lung cancer	A randomized phase II study of ITZ and pemetrexed in patients with previously treated non-squamous non-small cell lung cancer	NCT00769600	II	Drug: ITZ combined with pemetrexed	Total life expectancy (up to 3 years); progression free survival, as determined by the number of days in a year without any disease progression; the number of individuals with solid tumors who had a partial response, stable illness, or progressing disease as determined by response evaluation criteria (up to 3 years) pharmacokinetics of ITZ taken orally	Terminated
				Drug: single agent pemetrexed		
Non-small cell lung cancer	Phase 0 pharmacodynamic study of the effects of ITZ on tumor angiogenesis and the hedgehog pathway in early-stage non-small cell lung cancer	NCT02357836	Early phase I	ITZ 600 mg	Variations from baseline in the density of tumor tissue microvessels	Completed
Prostate cancer	A randomized phase II clinical trial of two dose-levels of ITZ in patients with metastatic castration-resistant prostate cancer	NCT00887458	II	ITZ 200 mg, ITZ 300 mg	The percentage of individuals with metastatic colorectal cancer (CRPC) who do not have PSA progression after 24 weeks	Completed
Breast cancer	A pilot trial of ITZ pharmacokinetics in patients with metastatic breast cancer	NCT00798135	Not applicable	ITZ 200 mg	Pharmacokinetics of oral ITZ	Completed
Prostate adenocarcinoma	Hedgehog inhibition as a non-castrating approach to hormone sensitive prostate cancer: a phase II study of ITZ in biochemical relapse	NCT01787331	II	ITZ 300 mg	The proportion of patients whose serum PSA drops by 50% or more after 12 weeks	Completed
Basal cell carcinoma skin cancer	Pilot biomarker trial to evaluate the efficacy of ITZ in patients with basal cell carcinomas	NCT01108094	II	ITZ 400 mg, ITZ 200 mg	After one month of treatment, the percentage change in the Ki67 tumor proliferation biomarker	Completed
Neoplasm metastasis	A phase 1, multicenter, open-label, single sequence crossover study to evaluate drug–drug interaction potential of OATP1B/CYP3A inhibitor on the pharmacokinetics of DS-8201a in subjects with HER2-expressing advanced solid malignant tumors	NCT03383692	I	Drug: DS-8201a and ritonavir	*C*_max_ after DS-8201a and ritonavir/ITZ treatment	Completed
				Drug: DS-8201a and ITZ		
Advanced solid tumors	A study to evaluate the effect of ITZ and rifampin on the pharmacokinetics of talazoparib in patients with advanced solid tumors	NCT03077607	I	Drug: talazoparib	AUC_0–last_ measurable concentration of talazoparib: alone and in combination with ITZ; AUC_0–inf_ of talazoparib: alone and in combination with ITZ; *C*_max_ of talazoparib: alone and simultaneously	Completed
				Drug: ITZ		
				Drug: rifampin		
Advanced solid tumors, relapsed/refractory lymphoma	A phase 1 study to evaluate the effect of ITZ, a strong CYP3A inhibitor, on the pharmacokinetics of alisertib (MLN8237) in adult patients with advanced solid tumors or relapsed/refractory lymphoma	NCT02259010	I	Drug: alisertib	AUC_0–last_ measurable concentration of alisertib in presence and absence of ITZ; AUC_0–inf_ of alisertib in presence and absence of ITZ; and *C*_max_ of alisertib between the two	Completed
				Drug: ITZ		
Acute myeloid leukemia	PKC412 in participants with acute myeloid leukemia or with myelodysplastic syndrome (CPKC412A2104 core); and PKC412 in participants with acute myeloid leukemia or with myelodysplastic syndrome with either wild type or mutated FMS-like tyrosine kinase 3 (FLT3) (CPKC412A2104E1 and CPKC412A2104E2)	NCT00045942	I	Drug: ITZ	Quantity of individuals who exhibited the best clinical response (CR, PR); percentage of phospho-FLT3 that has decreased from the baseline; the total number of participants that responded clinically	Completed
				Drug: PKC412		
Solid tumor	A study to evaluate the amount of drug that becomes available in the blood circulation when savolitinib is administered alone and in combination with ITZ	NCT04121910	I	Drug: savolitinib	Comparing the geometric averages of the test treatment (savolitinib + ITZ) to the reference treatment (savolitinib alone), the area under the plasma concentration–time curve from time zero to infinity (AUC) ratios	Completed
				Drug: ITZ		
Advanced solid malignancies	Drug–drug interaction study with AZD5305 and ITZ in patients with advanced solid malignancies	NCT05573724	I	Drug: AZD5305	Part A: area under the concentration–time curve from time zero to infinity (AUCinf)	Completed
				Drug: ITZ		
Advanced solid malignancies	A study to assess the effects of ITZ, rifampicin, and omeprazole on pharmacokinetics of adavosertib	NCT04959266	I	Drug: adavosertib	Summary of adavosertib plasma concentrations with time	Terminated
				Drug: ITZ		
				Drug: rifampicin		
				Drug: omeprazole		
Esophageal squamous cell carcinoma	CCRT with ITZ in locally advanced squamous esophageal cancer	NCT04481100	II	Drug: ITZ	Four to eight weeks after RT was finished, the objective response rate (ORR) was assessed and documented using RECIST, version 1.1	Unknown
Solid tumor	Study to investigate the effect of rifampin and ITZ on the action of pamiparib in participants with cancer	NCT03994211	I	Drug: pamiparib 60 mg	Maximum observed concentration (*C*_max_) of pamiparib in plasma for part A	Completed
				Drug: pamiparib 20 mg		
				Drug: ITZ		
				Drug: rifampin		
				Drug: pamiparib		
Metastatic patients with triple negative or HR+ breast cancer, or hormone sensitive prostate cancer	A study to assess how ITZ affects the uptake and elimination of capivasertib in the body	NCT04712396	I	Drug: capivasertib	Assessment of AUCinf of capivasertib	Completed
				Drug: ITZ		
Advanced malignancies	Study to describe the interaction between tazemetostat and ITZ and between tazemetostat and rifampin in participants with advanced cancer	NCT04537715	I	Drug: tazemetostat	Part 1: area under the plasma concentration–time curve from time 0 to 12 hours of quantifiable concentration (AUC_0–12 h_) of tazemetostat	Completed
				Drug: ITZ		
				Drug: tazemetostat		
				Drug: rifampin		

a ClinicalTrials.gov [Internet]. Available from: https://clinicaltrials.gov/search?cond=Cancer&intr=itraconazole&start=2018-01-01_2023-10-19&firstPost=2018-01-01_2023-10-19&page=1, accessed May 12, 2026.

## Comparative analysis of drug delivery systems for itraconazole

**Fig. 4:**
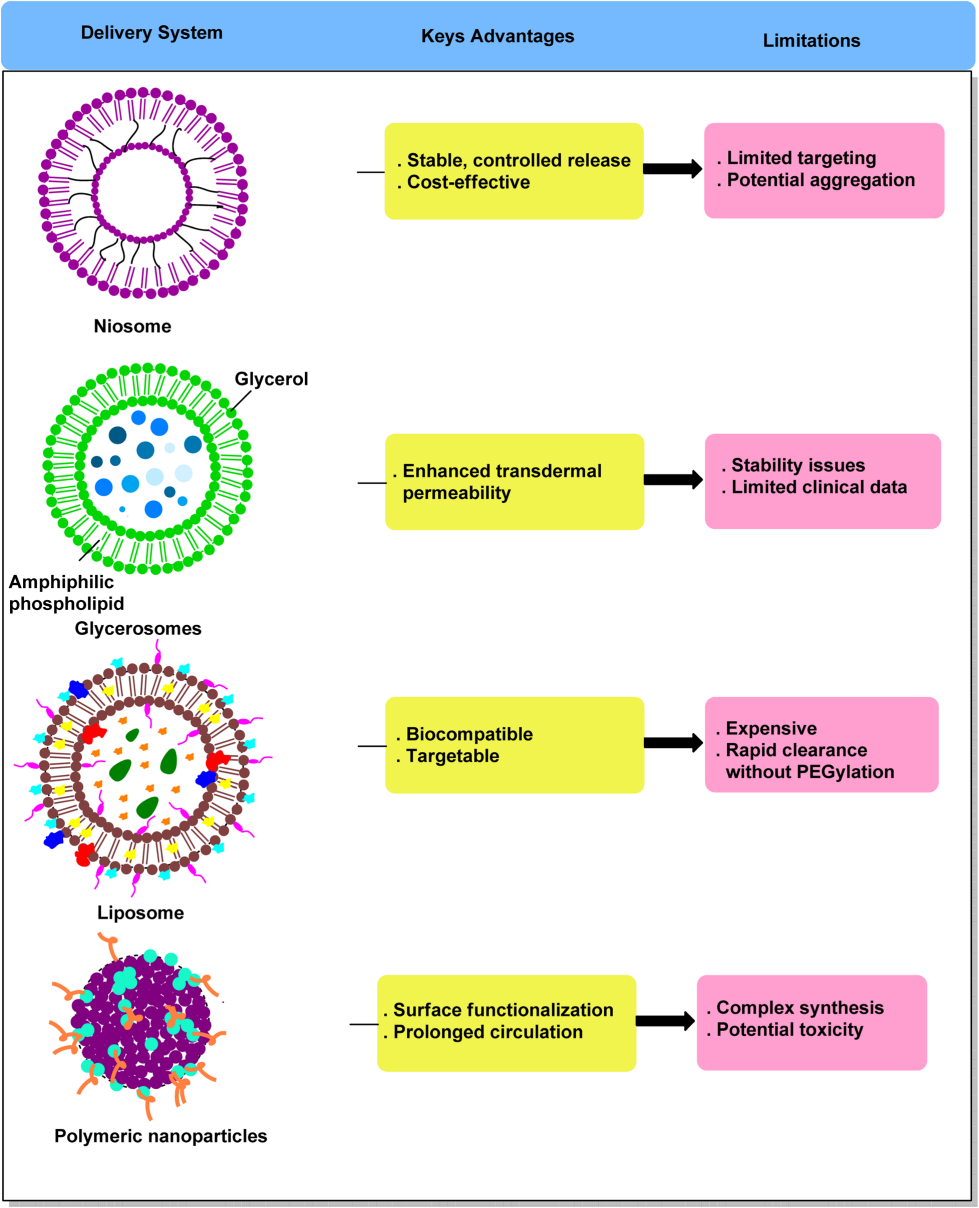
Comparative overview of itraconazole delivery systems.

The development of sophisticated drug delivery technologies, such as niosomes, glycerosomes, liposomes, and polymeric nanoparticles, has been spurred by ITZ 's limited aqueous solubility and inconsistent oral bioavailability. Glycerosomes increase transdermal permeability because of its glycerol concentration, whereas niosomes provide improved stability and regulated release. While polymeric nanoparticles provide surface functionalization and extended circulation, liposomes offer biocompatibility and tailored distribution. Despite these benefits, there are still few direct comparisons between these methods. To inform future translational research, a systematic assessment that takes into account encapsulation efficiency, release profile, bioavailability, toxicity, and anticancer activity is crucial^47–49^ (Fig. 4).

## Review of recent research on ITZ as a potential therapeutic agent for the treatment of cancers (2021–2025)

For almost 35 years, ITZ, has been utilized in therapeutic settings. Numerous studies conducted recently have shown that ITZ has anti-cancer qualities and has already been evaluated in cancer treatment^50–52^ for non-small cell lung cancer,^38^ gastric cancer,^36^ prostate cancer,^40^ and basal cell carcinoma.^39^ ITZ is a promising anticancer drug that can be used either alone or in combination with chemotherapy, according to preclinical and clinical studies.^20^ This section of the review focuses on the most recent research on ITZ as a possible therapeutic strategy for cancer treatment (2021–2025).

## Lung cancer

Mohamed, Asmaa Waheed, *et al.*^53^ conducted a randomized controlled trial (RCT) to evaluate the addition of ITZ to platinum-based chemotherapy in patients with advanced NSCLC. Patients received ITZ (200 mg daily for 21 days per cycle) plus chemotherapy or chemotherapy alone. The ITZ group demonstrated a significantly higher overall response rate (ORR) and longer progression-free survival (PFS), whereas no significant improvement in overall survival (OS) was observed. Treatment was generally well tolerated, with manageable toxicity. These findings suggest that ITZ may enhance short-term treatment efficacy when combined with standard chemotherapy. These findings are consistent with other drug-repurposing studies in NSCLC, where ITZ shows anti-tumor activity; however, the lack of OS benefit suggests its clinical impact may be limited to improving ORR and PFS rather than extending survival.

**Fig. 5:**
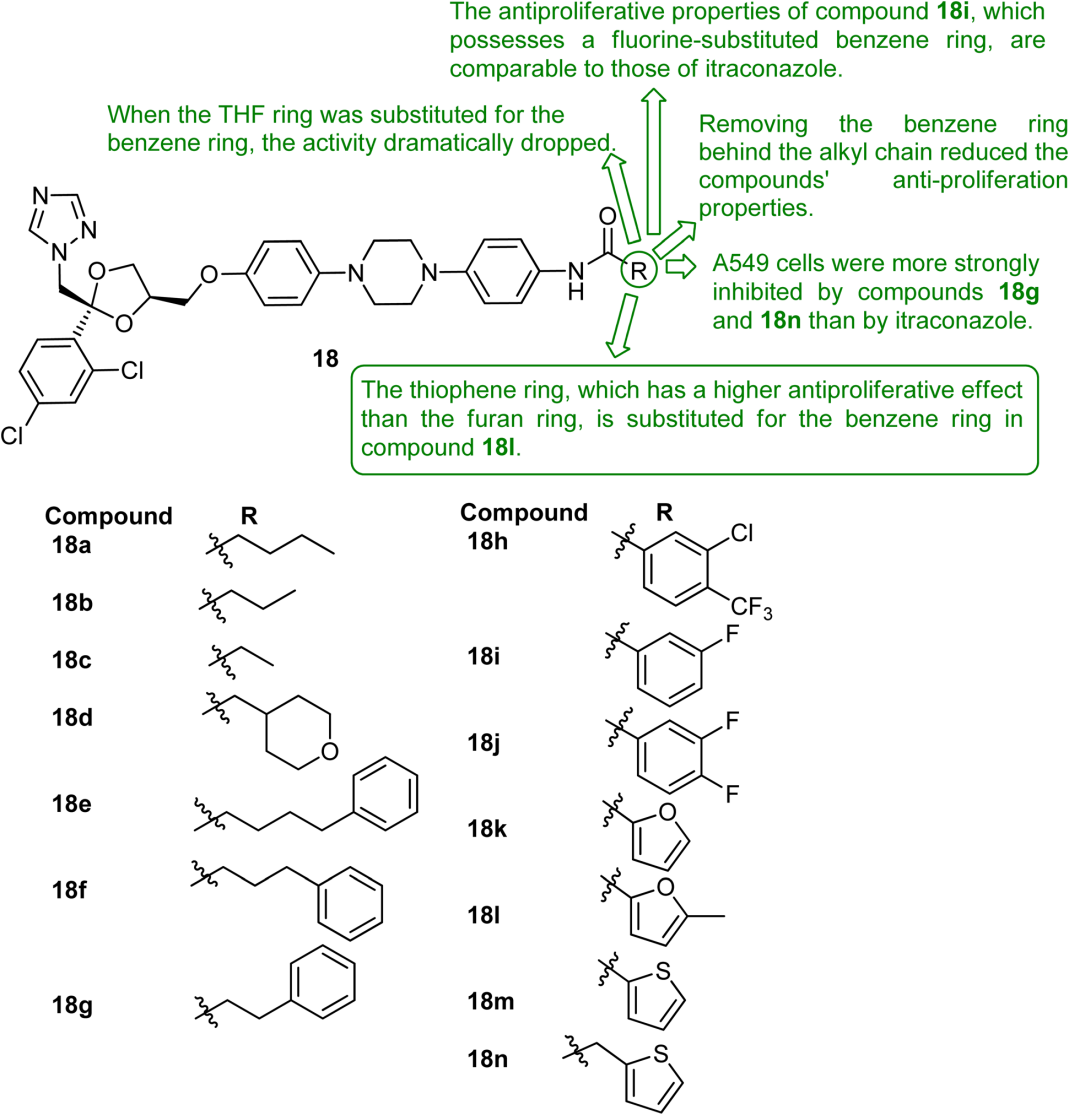
The chemical structure of a number of derivatives was created and produced using ITZ, an inhibitor of the hedgehog signal channel.

The mechanism of the Hh signaling pathway and the anticancer effects of hedgehog inhibitors were investigated in this study, with ITZ serving as a key reference compound. Novel ITZ derivatives were designed and synthesized, and their anticancer activity was evaluated in NSCLC A549 cells. Among them, compounds 18g and 18n exhibited stronger anti-proliferative and Hh pathway inhibitory effects than ITZ and showed greater activity in A549 cells than in HepG2 liver cancer cells. Mechanistic assays demonstrated inhibition of colony formation, induction of apoptosis, increased reactive oxygen species, and mitochondrial dysfunction, suggesting that structural modification of ITZ may enhance its anticancer potency against NSCLC (Fig. 5).^54^ While clinical studies have evaluated ITZ as a repurposed agent, this preclinical work suggests that structural modification of ITZ may yield more potent hedgehog pathway inhibitors with enhanced activity against NSCLC.

This study examined the potential physiological effects of two repurposed drugs, ITZ and cilostazol, in the context of anaplastic lymphoma kinase (ALK)-positive NSCLC. The authors proposed that adding ITZ and cilostazol to lorlatinib may enhance therapeutic efficacy by targeting multiple resistance pathways. ITZ inhibits P-glycoprotein (P-gp), CYP3A4, Wnt signaling, and hedgehog signaling, potentially increasing lorlatinib brain penetration and disrupting tumor growth signaling. Cilostazol, a phosphodiesterase-3 inhibitor, may reduce platelet-mediated tumor support by decreasing platelet aggregation and growth factor release. Given the poor prognosis of metastatic ALK-positive NSCLC and the anticipated safety of this combination, the study supports clinical evaluation of lorlatinib augmented with ITZ and cilostazol to delay resistance.^55^ Unlike studies focusing on ITZ as a direct anticancer agent, this work emphasizes its role in overcoming targeted therapy resistance through modulation of drug transport and oncogenic signaling pathways.

This study investigated glycerosomes (GLY), glycerol-based nanovesicles, as a novel pulmonary delivery system for ITZ. To enhance anticancer efficacy and prolong local drug residence, the vesicles were functionalized with hyaluronic acid (HA-GLY). *In vitro* and *in vivo* findings demonstrated that HA-GLY improved ITZ cytotoxicity, lung deposition, and biodistribution, likely through CD44 receptor-mediated targeting and enhanced permeability and retention (EPR) effects. Intratracheal administration of this nanoplatform enhanced local pharmacological activity, reduced dosing frequency, and may minimize systemic toxicity, suggesting a promising strategy for targeted lung cancer therapy.^56^ Unlike conventional systemic administration of ITZ, this nanotechnology-based approach improves pulmonary targeting and local drug retention, highlighting the potential of advanced delivery systems to enhance the therapeutic index of repurposed agents.

Zheng, Hongmei, *et al.*^57^ demonstrated that ITZ enhances the efficacy of osimertinib in osimertinib-resistant NSCLC. The combination inhibited tumor cell proliferation and migration, induced apoptosis, and suppressed tumor growth by promoting proteasomal degradation of sonic hedgehog (SHH), thereby inactivating the SHH/DUSP13B/p-STAT3 and hedgehog pathways and downregulating c-Myc. Mechanistic analyses revealed that DUSP13B modulates STAT3 phosphorylation, and SHH overexpression partially restores resistance, highlighting the central role of ITZ in reversing acquired osimertinib resistance. These findings provide a mechanistic rationale for combining ITZ with osimertinib in resistant NSCLC. Compared with studies using ITZ as a direct anticancer agent, this work emphasizes its ability to overcome targeted therapy resistance by modulating the SHH/DUSP13B/p-STAT3 signaling axis, reinforcing ITZ's potential in combination strategies for NSCLC.

In this work, an inhalable formulation of ITZ using nanoparticles-in-microparticles (NIM) stabilized with polyvinyl alcohol 500 (PVA) was developed and evaluated for pulmonary delivery. Pharmacokinetic studies in Sprague-Dawley rats demonstrated improved distribution of ITZ in lung tissues and bronchoalveolar lavage fluid (BALF), enhanced survival, reduced *Aspergillus fumigatus* growth, and decreased galactomannan levels. Compared with oral administration, pulmonary delivery reduced hepatotoxicity markers (ALP, ALT) and increased local drug retention. These findings highlight the potential of PVA-based NIMs as a safe and effective inhalable therapy for pulmonary fungal infections.^58^ Compared with conventional oral ITZ, this nanotechnology-based inhalable approach improves pulmonary targeting, minimizes systemic toxicity, and underscores the advantages of advanced delivery systems in respiratory medicine.

## Skin cancer

The purpose of this research is to examine the molecular mechanism of ITZ's therapeutic potential in cutaneous squamous cell carcinoma (cSCC). The CCK-8 assay and the clone formation assay were used to investigate ITZ's anti-proliferation impact. ITZ prevented cSCC cells from proliferating, caused apoptosis, and stopped their cell cycle. Acyl-CoA synthetase long-chain family member 4 (ACSL4) and 3-hydroxy-3-methylglutaryl-CoA synthase 1 (HMGCS1) were shown to be markedly increased in A431 cells treated with ITZ, according to a combined transcriptome and proteomic investigation. In A431 cells, HMGCS1 knockdown reversed ITZ's antiproliferative effects. The dual-luciferase test demonstrated that ITZ could increase the transcription of HMGCS1. In A431 cells, HMGCS1 suppression reduced ACSL4 expression. ITZ enhanced the amount of reactive oxygen species (ROS), lipid peroxidation, and iron buildup. Furthermore, ITZ treatment inhibited the formation of tumors in mice that had A431 genes. This study demonstrated that ITZ suppresses cSCC growth by controlling the HMGCS1/ACSL4 axis.^59^

This work investigated how ITZ induces autophagy-mediated apoptosis in melanoma cells. SQSTM1 was found to be the main target of ITZ using the probabilistic matrix factorization (PMF) machine learning algorithm. By causing autophagy-mediated apoptosis and G1 phase arrest in A375 and A2058 cells, ITZ suppressed the growth of melanoma cells. Additionally, ITZ interfered with the activation of the recombinant human Sonic Hedgehog (rhSHH), a hedgehog agonist, and inhibited hedgehog signaling. ITZ dramatically slowed the growth of tumors in the A375 and A2058 xenograft models *in vivo*.^60^

An auspicious method in the current work would be the nanovehiculation and optimization of the repositioned ITZ using ascorbyl palmitate (AP) aspasomes. Repurposing current medications to combat malignant neoplasms could be a positive step toward successful cancer remediation. Additionally, a cream was made with the optimized aspasomes, and their skin deposition was monitored. Aspasomal cream's *in vivo* effectiveness was evaluated using a mouse subcutaneous Ehrlich cancer model. The improved aspasomes demonstrated good colloidal stability, >95% ITZ entrapment, nano size (67.83 ± 6.16 nm), and negative charge (−79.40 ± 2.23 mV). With an IC_50_ of 5.3 ± 0.27 µg mL^−1^, AP demonstrated significant antioxidant ability and advanced ITZ cytotoxicity against A431 cells. The aspasomal cream, which shown 62.68% tumor weight decrease, confirmed spreadability, considered skin penetration, and enhanced *in vivo* anticancer competence, was an alluring advantage. These cooperative tumor probes lay the groundwork for innovative clinical translation and business development.^61^

The goal of this research was to create ITC nanoformulations that were more effective against cancer. Due to the possibility for oral treatment of skin cancer with ITZ (ITC), an antifungal medication having anticancer properties. As bioactive additions, lipid nanocapsules (LNC) were created, either untreated (ITC/LNC) or modified with the lipopeptide biosurfactant surfactin (ITC/SF-LNC) or the amphiphile miltefosine (ITC/MF-LNC). LNC formulations demonstrated sustained ITC release, a small diameter (42–45 nm), and a high ITC entrapment efficiency (>98%). The LNC formulations significantly increased the ITC anticancer activity and selectivity for cancer cells, and a synergistic ITC–amphiphile interaction improved the combination performance, according to cytotoxicity experiments conducted using malignant SCC 9 cells and normal human fibroblasts (NHF). ITC/MF-LNC and ITC/SF-LNC significantly increased the tumor growth suppression and skin architecture recovery of intradermal tumor-bearing mice treated with ITC nanoformulation gels as opposed to ITC and 5-FU gels. LNC formulations had a greater suppressive effect on cytokeratins than 5-FU, and they considerably reduced tumoral production of Ki-67 and cytokeratin proliferative proteins. These results offer fresh proof that low-risk skin carcinogenesis can be effectively treated topically using a variety of strategies that include medication repurposing, nanotechnology, and bioactive amphiphiles as formulation-enhancing additives.^62^

Fan, Ni, *et al.*^63^ examined how melanoma progression is mediated by ERK signaling and the role of ITZ-induced 5′-monophosphate AMPK alpha (AMPKα). RT-qPCR and western blot were used to measure the amount of AMPKα in melanoma tissues and cells. According to the TCGA database, a survival analysis of melanoma patients based on their AMPKα expression level was conducted. Assays for wound healing, colony formation, CCK-8, and Transwell were used to investigate the invasion, migration, and proliferation of melanoma cells. An *in vivo* study of ITZ's impact on tumor growth was conducted using a xenograft tumor model. The tissues and cells of melanoma showed decreased levels of AMPKα mRNA and protein. A poor prognosis was indicated by low AMPKα expression. By upregulating AMPKα, ITZ inhibited the migration, invasion, and proliferation of melanoma cells. ITZ reduced ERK signaling in melanoma cells while activating AMPK signaling. When ERK signaling was activated, ITZ's impact on melanoma cellular processes was reversed. Furthermore, *via* blocking ERK signaling, ITZ-induced AMPKα prevented the formation of melanoma tumors *in vivo*. Melanoma development is inhibited by ITZ-induced AMPKα through ERK signaling suppression.

In the current study, ITZ-loaded transferosomes were created, optimized, and repurposed for skin cancer using the Quality by Design (QbD) methodology. For formulation optimization, a mix of optimization design and screening was employed. The improved formulation has an entrapment effectiveness of 64.11 ± 3.75%, a zeta potential of −47.80 ± 3.66, a particle size of 192.37 ± 13.19 nm, and a PDI of 0.41 ± 0.03. ITZ-encapsulated transferosomes have a higher and longer-lasting release than pure medications, according to *in vitro* release experiments. Transferosome penetration and retention in the skin are more obvious than in the drug, according to *ex vivo* drug penetration and retention tests. ITZ-encapsulated transferosomes are nearly twice as effective against the A375 cell line as the medication itself, according to the cell viability research. Combining screening and optimization strategies resulted in the successful preparation and optimization of ITZ encapsulated transferosomes. ITZ-loaded transferosomes may help manage melanoma in addition to conventional treatments, according to this study's findings based on *ex vivo* and cell line investigations.^64^

## Breast cancer

In order to enhance the autophagy-modulated immune response against breast cancer metastasis and recurrence, this work has created a simple and reliable chemophotothermal nanoplatform that combines ITZ and IR820 (IC/IR820 NPs). (ROS)-mediated apoptosis and autophagic death are two ways that the IC/IR820 NPs have an improved therapeutic effect on breast cancer. Additional testing in a mouse model has demonstrated the remarkable effects of IC/IR820 NPs in preventing tumor spread and triggering immunity to stop tumor recurrence. Mechanistically, ITZ may stimulate dendritic cell activation and tumor cell antigen presentation *via* autophagy, eliciting an immunological response that works in concert with the immune response produced by photothermal therapy to prevent tumor recurrence. Excellent therapeutic efficacy is achieved by co-assembling ITZ and IR820 into a single, robust, and minimalist nanoplatform, indicating that this approach may be used as a substitute for current clinical breast cancer therapy techniques.^65^

The current study examined the possible use of ITZ in combination with rapamycin for the treatment of triple negative breast cancer (TNBC), with a focus on medications that target the mTOR signaling system. To assess the impact of ITZ and rapamycin together on MDA-MB-231 and BT-549 TNBC cells, CCK-8, colony formation, and Transwell assays were performed. TNBC cell motility and proliferation were found to be synergistically inhibited. However, using ITZ and rapamycin together did not increase apoptosis. ITZ and/or rapamycin stopped cells in the G0/G1 phase and stopped the G1/S phase transition, according to a flow cytometry investigation. G0/G1 phase arrest was consistent with decreased cyclin D1 protein levels, particularly when ITZ and rapamycin were combined. As a result of their synergistic ability to stop cells in the G0/G1 phase of the cell cycle rather than causing apoptosis, ITZ and rapamycin together represent a promising treatment approach for patients with TNBC.^66^

In this study, ITZ and endothelial growth factor (VEGF) siRNA were co-loaded into composite nanoparticles to examine their anti-angiogenesis effectiveness and synergistic anticancer effect against breast cancer. The nanoparticles exhibited a good stability and drug release profile *in vitro*, along with an appropriate particle size (117.9 ± 10.3 nm) and weak positive surface charge (6.69 ± 2.46 mV). Additionally, the nanoparticles were able to effectively escape endosomes and decrease cell growth and death *in vitro*. The co-loaded ITZ-VEGF siRNA NPs could successfully reduce tumor development with low toxicity and adverse effects, and *in vitro* and *in vivo* tests demonstrated that the nanoparticles could promote the silencing of VEGF-related expressions as well as anti-angiogenesis efficacy. When combined, the as-prepared delivery vehicles provide a straightforward and secure nano-platform that enhances the antitumor efficacy of ITZ and VEGF siRNA, repositioning the generic medication as a strong contender for antitumor therapy.^67^

Park, Jung Min, *et al.*^68^ aimed to challenge trastuzumab resistance in HER2-positive breast cancer by examining the anti-cancer effects of ITZ on cell proliferation, apoptosis, autophagy, and breast cancer stem cell-like characteristics. ITZ's *in vitro* effects on the trastuzumab-resistant cell line JIMT-1 were investigated in terms of autophagy, apoptosis, cell viability, and its influence on cancer stem cells. The anti-tumor efficacy of ITZ was investigated by implanting trastuzumab-resistant JIMT-1 cells to create xenografts, similar to an *in vivo* experimental paradigm. ITZ treatment markedly induced apoptosis and greatly inhibited the proliferation of JIMT-1 cells. In JIMT-1 cells, ITZ decreased the levels of phosphorylation of p185HER2 and truncated-p95HER2. Furthermore, the results of the rise in LC3 I/II and the fall in Beclin-1 and p62 levels following ITZ exposure confirmed that ITZ also triggered autophagy. Crucially, ITZ successfully eliminated populations that resembled cancer stem cells in addition to killing tumor cells that were growing. The ALDH1 activity assay and FACS analysis of the CD44+/CD24− stem-like phenotype in JIMT-1 cells were performed in order to clarify the eradication of the cancer stem-like population by ITZ. Consequently, there was a marked decline in ALDH1 activity and CD44+/CD24− stem-like populations, indicating that stem-cell-like populations were compromised. The physiological significance of *in vitro* observations was validated using an *in vivo* mouse model. ITZ treatment resulted in a marked reduction of tumor burden and growth in trastuzumab-resistant xenografts, along with significant downregulation of HER2, ALDH1, and microvessel density and a sharp drop in p62 *in vivo*. There was no statistically significant difference in the serum levels of ALT, AST, and BUN between the groups that received vehicle and ITZ, indicating that there was no harm to the liver or kidneys from ITZ. This study has shown that ITZ, an FDA-approved antifungal medication, targets cancer stem-like characteristics, suppresses HER2 signaling, and induces autophagy to produce anti-tumor action in trastuzumab-resistant HER2-positive breast cancer. These results lend credence to the idea that ITZ may represent a novel therapeutic strategy for HER2-positive breast tumors that are resistant to trastuzumab.

## Colon cancer

Through the use of the MTT assay, this study examined the anti-cancer effects of ITZ and paclitaxel both *in vitro* and *in vivo*, as well as their anti-cancer synergistic impact in HT-29 tumor-bearing nude mice and YM-1 and HT-29 cell lines. For additional evaluation, a histopathological experiment was conducted. Additionally, ITZ's inhibitory effect on P-glycoprotein (P-gp) was assessed by ^99m^Tc-MIBI uptake and specific *in vivo* biodistribution. Myocardial perfusion imaging agent ^99m^Tc-MIBI is a helpful radiotracer in the diagnosis of some malignancies, and P-gp regulators alter the accumulation of ^99m^Tc-MIBI in the liver and tumor. In both *in vitro* and *in vivo* xenograft models, the data presented demonstrated experimental support for the possible anticancer effect of paclitaxel and ITZ, as well as the two in combination, in human colon cancer. These findings might help oncologists create a new chemotherapy regimen for colon cancer that includes paclitaxel and ITZ.^69^

HT-29 tumor-bearing nude mice were used in this investigation to test the effectiveness of ITZ as a P-gp inhibitor and its therapeutic synergistic connection to paclitaxel through ^99m^Tc-MIBI accumulation. For additional evaluation, histopathological screening and *in vitro* tests were conducted. *In vitro* accumulation of ^99m^Tc-MIBI in ITZ-receiving dishes increased as a result of ITZ's effective inhibition of P-gp-mediated efflux. The co-administration of paclitaxel and ITZ considerably increased the paclitaxel's *in vitro* cytotoxicity impact in HT-29 cell-containing ITZ + paclitaxel wells. ITZ, paclitaxel, and ITZ + paclitaxel-treated mice had tumor volumes that were roughly 36.21, 60.02, and 73.3% smaller than the control group. At the conclusion of the treatment period, the nude mice co-treated with paclitaxel and ITZ demonstrated a decrease of tumor growth of roughly 33.31 percent in comparison to the paclitaxel group. Additionally, the biodistribution result demonstrated that, in comparison to the control and paclitaxel groups, the mean tumor radioactivity accumulation increased when ITZ and paclitaxel were administered together. The ID% of cardiac and hepatic tissue decreased when paclitaxel was administered alone, while ^99m^Tc-MIBI accumulation in these organs increased when ITZ and paclitaxel were administered together. The biodistribution results were also validated by the histopathology data. These results demonstrated that although paclitaxel or ITZ by themselves are effective against HT-29 human colorectal cancer, co-administration of the two drugs can have a synergistic anti-tumor impact. Additionally, ^99m^Tc-MIBI is a useful radiotracer for tracking how well multidrug-resistant (MDR) tumors are responding to treatment.^70^

Shen, Pei-Wen, *et al.*^71^ examined a large amount of data from the Taiwanese National Health Insurance Research Database pertaining to patients who were ITZ-treated for colon cancer between January 2011 and December 2015. ITZ's underlying molecular pathways in autophagy-induced cell death were also examined. The findings showed that patients with colon cancer treated with ITZ had a considerably greater 5 year survival rate. Furthermore, ITZ caused cleaved caspase-3 expression and G1 cell cycle arrest in COLO 205 and HCT 116 cells, while also reducing viability and cell colony formation. Notably, ITZ increased the expression of p62 and LC3B, which in turn caused autophagy. The viability of ITZ-treated COLO 205 and HCT 116 cells significantly increased after LC3 knockdown. When combined, the study's findings imply that ITZ may benefit colon cancer patients, and that the underlying molecular mechanisms of the drug may be linked to the triggering of autophagic cell death.

This study used targeted organoid sequencing data to analyze FDA-approved medications and discovered that the antifungal medication ITZ may be able to treat colorectal cancer tumors. ITZ's impact and mechanism on colorectal cancer tumors, however, have not been studied. Single-cell RNA sequencing was carried out on tumor samples from four mice treated with or without ITZ using a cell line-derived xenograft model in tumor-bearing mice. Between the two groups, there was a substantial difference in the percentage of cell populations and gene expression profiles. According to this study, ITZ may prevent tumor growth and glycolysis. This study discovered that CEBPB was a novel target for ITZ and that, by preventing Enolase 1 (ENO1) expression, Enhancer Binding Protein Beta (CEBPB) silencing might suppress advanced colorectal cancer (CRC) glycolysis and tumor development. This research showed that CEBPB was a novel target for ITZ and that, by preventing ENO1 expression, CEBPB silencing might suppress CRC glycolysis and tumor development. Clinical study revealed that individuals with colorectal cancer had clearly higher levels of CEBPB expression, which was linked to a lower chance of survival. In conclusion, gene expression profiles and cell composition were altered by ITZ treatment. Through the CEBPB–ENO1 axis, ITZ suppressed tumor development and cell glycolysis. This work provided a theoretical foundation for CRC targeting/combination therapy by illuminating a novel energy metabolism mechanism for ITZ on tumor growth in CRC.^72^

This research examined the anticancer effects of compounds diosgenin and ITZ on colon cancer cells as well as the efficacy of drug delivery *via* a niosomal drug delivery system. A significant step towards efficient administration was demonstrated by the successful manufacture and characterization of niosomes as drug delivery vehicles, which demonstrated great encapsulation efficiency and successful binding within the hydrophobic interior of the niosomes. Non-ionic surfactants, cholesterol, and the appropriate compound were utilized to prepare each compound-loaded niosome. The process involved sonication after thin film hydration with a rotary evaporator. The average size of the niosomes loaded with ITZ was 343.8 nm, while the niosomes encased in diosgenin were 194.3 nm, according to the dynamic light scattering. ITZ and diosgenin were shown to have encapsulation efficiencies of 72.4% and 69%, respectively, for their niosomes. When given to HCT116 colorectal cancer cell lines, cytotoxicity tests showed that the chemicals ITZ and diosgenin had IC_50_ values of 53.84 µM and 45.8 µM, and the corresponding loaded niosomes had IC_50_ values of 36.58 µM and 59.58 µM. The compound's effectiveness is further confirmed by cytotoxicity experiments, which show a marked reduction in cell death upon encapsulation, indicating improved safety and fewer adverse effects. These results give hope for better treatment outcomes with fewer side effects by paving the path for the bioactive chemicals ITZ and diosgenin to continue preclinical and clinical development in colon cancer therapy.^73^

## Cervical and ovarian cancer

This study used the anticancer drug ITZ to identify a novel therapeutic target. Compared to other cancer cell lines, the human cervical squamous carcinoma cell line (CaSki) was utilized since ITZ affects it the most. The metabolic pathways of potential specialized pro-resolving mediators (SPMs) were targeted by inhibitors and ITZ in cell growth tests. As potential SPMs, resolvin E3, resolvin E2, prostaglandin J2 (PGJ2), delta-12-PGJ2, and maresin 2 were found. The 12/15-lipoxygenase inhibitor lessened the inhibitory effect of ITZ. This enzyme is involved in the conversion of 18-hydroxy-eicosapentaenoic acid to resolvin E3. Treatment with ITZ was unaffected by inhibition of the PGJ2 metabolic pathway. Resolving E3 is part of the metabolic pathway of SPMs, which may be ITZ's anticancer target.^74^

Isono, Roze, *et al.*^75^ looked at how ITZ affected the lipid content of the membrane and inhibited the movement of cholesterol in CaSki cervical cancer cells. Cervical cancer CaSki cells were cultivated with ITZ and examined using filipin staining and confocal microscopy to look into the effects of the drug on cholesterol trafficking. The distribution of cholesterol in the intracellular compartments following ITZ treatment was comparable to that observed following treatment with U18666A (cholesterol transport inhibitor), according to filipin staining. Phosphatidylserine levels in CaSki cells significantly decreased, but lysophosphatidylcholine levels increased, according to liquid chromatography/mass spectrometry (LC/MS) research. ITZ changed the phospholipid makeup and prevented cholesterol from moving. ITZ's anticancer action may be enhanced by modifications to the cell membrane.

This study presented the evidence that five medications used in general medicine, known as the EC5 regimen, prevent the progression of endometrial cancer. Alendronate, an osteoporosis treatment; celecoxib, an analgesic; ITZ, an antifungal; ramelteon, a sleep aid; and simvastatin, a cholesterol-lowering medication, were all used in the EC5 regimen. These medications typically have little side effects that patients may tolerate. Although there is a good case for using the EC5 regimen as an adjuvant to treat EC, there is insufficient evidence of its safety or effectiveness.^76^

Tumor-associated macrophages (TAMs) and their role in the tumor-agnostic pathway were examined in this study. Human monocyte leukemia cell line (THP-1) was used to create M1 and M2 macrophages, and their morphological characteristics were assessed. Western blots and the enzyme-linked immunosorbent test were used to assess cell membrane antigens and secreted proteins, respectively. Using liquid chromatography and tandem mass spectrometry, the proteome profiling of cells was carried out and examined. Following the addition of M2 macrophage supernatant and during co-cultivation with M2 macrophages, with or without 10^−5^ M ITZ, the viability of cervical cancer cells (CaSki) was assessed. M1 macrophage co-culture decreased CaSki cell proliferation (*p* = 0.012), but M2 macrophage co-culture increased CaSki cell proliferation (*p* < 0.0001). After receiving ITZ for 24 hours, M2 macrophages transformed into M1-like cells and showed reduced expression of chemokine ligand 18 (CCL18) and cluster of differentiation 163 (CD163). For seven weeks throughout the ITZ treatment, the M1-like shape persisted, and following ITZ removal, it returned to its normal state. Proteomic examination of M2 macrophages treated with ITZ also revealed an M1-like signature, including increased amounts of proteins linked to tumor necrosis factor (TNF). Following ITZ treatment, CaSki cell growth was strongly decreased by both the M2 macrophage supernatant and the co-culture with M2 macrophages (each, *p* < 0.0001). ITZ exhibited TAM-mediated anti-cancer efficacy by suppressing cervical cancer cell proliferation and repolarizing M2 macrophages to M1 type.^77^

This research examined the spectrum of action of ITZ by testing a panel of 28 epithelial ovarian cancer (EOC) cell lines. A whole-genome drop-out genome-scale clustered regularly interspaced short palindromic repeats sensitivity test was conducted in two cell lines (TOV1946 and OVCAR5) in order to detect synthetic lethality when combined with ITZ. Accordingly, a phase I dose-escalation trial evaluating the combination of ITZ and hydroxychloroquine in patients with platinum-refractory EOC (NCT03081702) was carried out. They discovered a broad range of ITZ sensitivity in the EOC cell lines. The trans-Golgi network, late endosomes/lysosomes, and lysosomal compartments were found to be significantly involved in the pathway analysis; the autophagy inhibitor chloroquine exhibits similar pathways. This study then showed that in EOC cancer cell lines, ITZ and chloroquine exhibited a Bliss-defined synergy. Additionally, cytotoxic synergy was linked to chloroquine's capacity to cause functional lysosome malfunction. During the clinical trial, 11 patients were administered ITZ and hydroxychloroquine for at least one cycle. With the suggested phase II dosage of 300 and 600 mg twice daily, respectively, treatment was both safe and practical. There were no unbiased answers found. Limited pharmacodynamic influence was shown by pharmacodynamic assessments on successive biopsies. By altering lysosomal function, ITZ and chloroquine have synergistic efficacy and a strong anticancer effect *in vitro*. When the dosage was increased, the medication combination showed no clinical anticancer efficacy.^78^

## Pancreatic cancer

This study examined the effectiveness and side effects of first-line chemotherapy in the form of gemcitabine, nab-paclitaxel, oxaliplatin, and ITZ (GnPO-ITC) for patients with metastatic pancreatic cancer (mPC). The study included 81 patients (mean age = 64 years) with an Eastern Cooperative Oncology Group (ECOG) performance status of 0–1. The patients received ITZ (400 mg) every two weeks, and nabpaclitaxel (125 mg m^−2^), gemcitabine (1000 mg m^−2^), and oxaliplatin (85 mg m^−2^) on day 1. The peritoneum (*n* = 23,28%), distant lymph nodes (*n* = 24,30%), liver (*n* = 55,68%), and lungs (*n* = 18,22%) were among the patient's detected metastases. Within 28 days of starting chemotherapy, 15 patients (19%) had common hematological adverse effects of grade ≥3. Peripheral sensory neuropathy was the primary cause of therapy cessation among the respondents, accounting for 36 patients (44%). Sixty-four percent of patients responded to treatment overall [95% CI = 54–75%]. The median overall survival was 14.4 months (95% CI = 11.4–17.3 months), while the median progression-free survival was 8.3 months (95% CI = 6.8–9.8 months). 24 (46%) of the 52 responders had conversion surgery, however this did not increase survival (*p* = 0.279). In 71 (88%) of the patients, irinotecan was needed as a second-line treatment. 33 patients (41%) received hepatic arterial chemotherapy, whereas 27 patients (33%) received radiation. With controllable toxicity, the GnPO-ITC regimen shown encouraging efficacy in reducing the progression of the disease and enhancing overall survival.^79^

This work provided the information and justification for supplementing gemcitabine, nab-paclitaxel, the current standard cytotoxic chemotherapy for pancreatic ductal adenocarcinoma, with five generic nononcology medications from general medical practice. IPIAD used an old antimicrobial drug called pyrimethamine to treat malaria or toxoplasmosis, an old antifungal drug called ITZ, an old broad-spectrum antibiotic called azithromycin, an old antibiotic called dapsone, and an angiotensin receptor blocker (ARB) called irbesartan to treat hypertension. Through an analysis of specific growth driving mechanisms involved in pancreatic ductal adenocarcinoma and a comparison with comprehensive information on ancillary features of the IPIAD medications, it is possible to forecast the therapeutic benefit and limit growth of the cancer with this augmentation regimen. Half-measures won't slow the progression of metastatic pancreatic ductal adenocarcinoma (PDAC).^80^

Chacon-Fajardo, Diego, *et al.*^81^ described the intricate stromal pathways *via* which ITZ, an oral antifungal medication utilized in clinical settings, enhances the overall anti-tumor response in PDAC at the single-cell level. Following ITZ therapy *in vivo*, the pro-tumorigenic CD105+ cancer associated fibroblast (CAF) signature was significantly downregulated, according to single-cell RNAseq analysis of the genetically modified KPC model (LSL-KrasG12D; LSL-Trp53 R172H/+). Additionally, therapy had a significant impact on myofibroblasts (myCAFs), which cause the fibrotic PDAC desmoplasia, according to single-cell trajectory inference of CAFs subsets. Immunofluorescence studies also showed reduced collagen deposition and changed matrix remodeling in tumors treated with ITZ, which corroborated these findings. Significant positive changes in macrophage populations, such as the enrichment of M1-like pro-inflammatory macrophage signatures (confirmed by immunofluorescence), and positive signaling *via* Cxcl9-Cxcr3 and Tnf1rs-1a/1b ligand-receptors supported these potentially encouraging effects on the immune environment of PDAC. In an advanced *in vivo* model of pancreatic cancer, scRNAseq showed that dampening of regulatory T cell homing and functional potency signals within the pancreatic tumor microenvironment was linked to increased CD8+ T-cell infiltration after ITZ treatment. More importantly, this was associated with a significantly improved response to immune checkpoint blockade. In conclusion, these combined molecular and bioinformatic investigations offer a scientific justification for the combination of immunotherapy and ITZ in the treatment of pancreatic cancer.

This study assessed the connection between pancreatic cancer and the B3GALT5 enzyme using bioinformatics and *in silico* research. FDA-approved medications 6-AZA-UTP and ITZ were found to be putative inhibitors of the B3GALT5 enzyme using molecular docking research. According to biological testing on pancreatic cancer cell lines AsPC-1 and MIA PaCa-2, both substances markedly decreased cell viability. By reducing SSEA-3 expression, both medications successfully inhibited the activation of the B3GALT5 enzyme, according to flow cytometry data. Additionally, both substances demonstrated strong anti-tumor actions by causing pancreatic cancer cells to undergo apoptosis and preventing cell adhesion, colony formation, and migration. Notably, neither medication had any harmful or carcinogenic effects and showed good absorption, distribution, metabolism, excretion and toxicity (ADMET) profiles. According to studies, ITZ and 6-AZA-UTP can efficiently decrease the activity of the B3GALT5 enzyme, which suppresses tumors and prevents metastases. These results imply that ITZ or 6-AZA-UTP can both suppress the activity of the B3GALT5 enzyme and could be useful therapeutic alternatives for treating pancreatic cancer by repurposing existing drugs.^82^

## Esophageal cancer

This study aimed to clarify the mechanism of action of ITZ and ascertain its impact on esophageal cancer. In adenocarcinoma and esophageal squamous cell carcinoma cell lines, ITZ caused G1-phase cell-cycle arrest and suppressed cell proliferation. ITZ was found to decrease the phosphorylation of protein kinase AKT in OE33 esophageal cancer cells using an unbiased kinase array. Additionally, in esophageal cancer cells, ITZ reduced the phosphorylation of upstream PI3K, transcriptional expression of the upstream receptor tyrosine kinase HER2, and downstream ribosomal protein S6. HER2 is targeted by the tyrosine kinase inhibitor lapatinib, and siRNA-mediated HER2 knockdown also inhibited the development of cancer cells *in vitro*. In mice whose esophagi and tumors contained measurable amounts of ITZ and its main metabolite, hydroxy-ITZ, ITZ markedly suppressed the growth of OE33-derived flank xenografts. Xenografts from mice treated with ITZ showed lower levels of HER2 total protein and AKT and S6 protein phosphorylation than xenografts from mice treated with a placebo. ITZ reduced the expression of the HER2 total protein and the phosphorylation of the AKT and S6 proteins in tumors in patients with esophageal cancer in an early phase I clinical trial (NCT02749513). These findings showed that ITZ, in part by blocking HER2/AKT signaling, has strong antitumor effects in esophageal cancer.^83^

The study's goal was to find out if blocking the SHH pathway would stop Barrett's Esophagus (BE) from developing into esophageal cancer. ITZ's effectiveness was examined utilizing a surgical rat reflux model of Barrett's Metaplasia (BM). ITZ (treatment group; 200 mg kg^−1^) or saline (control group) intraperitoneal injections were administered weekly beginning 24 weeks after surgery. Compared to ITZ, which was found in 22 of 24 animals (91%), BM was found in 29 of 31 control animals (93%). EAC was substantially lower in control 10 of 31 (32%), compared to ITZ 2 of 24 (8%) (*P* = 0.033). ITZ reduced esophageal SHH levels compared to control (*P* = 0.12). Within 24 months of ablative treatment, esophageal tissue from patients with recurrent or persistent dysplastic BE showed strong expression of SHH and Indian hedgehog in distal BE compared to proximal squamous epithelium (odds ratio = 6.1 (95% CI: 1.6, 23.4) and odds ratio = 6.4 (95% CI: 1.2, 32.8)), respectively. In a preclinical animal model of BM, ITZ dramatically reduces SHH expression and EAC formation. Higher levels of SHH, Indian hedgehog, and bone morphogenic protein are expressed in BE tissue in humans compared to normal squamous esophageal epithelium.^84^

## Prostate cancer

Lima, Thiago S., *et al.*^85^ previously created two Docetaxel (DTX)-resistant prostate cancer cell lines, LNCaP^R^ and C4-2B^R^. These cell lines were developed from the androgen-dependent LNCaP cell line and the androgen-independent C4-2B sub-line, which were derived from the LNCaP lineage, respectively. An objective drug screen was used in this study to determine that the oral antifungal medication ITZ effectively re-sensitizes drug-resistant LNCaP^R^ and C4-2B^R^ prostate cancer cells to DTX treatment. ITZ can re-sensitize a variety of DTX-resistant cell types, including docetaxel-resistant breast cancer cells as well as cells derived from prostate cancer, including PC-3 and DU145. The expression of the ATP-binding cassette (ABC) transporter protein ABCB1, often referred to as P-glycoprotein (P-gp), is necessary for this action. ITZ binds firmly to the inward-facing form of ABCB1, blocking the transport of DTX, according to molecular modeling of ITZ bound to ABCB1. These findings implied that ITZ might offer a workable method for re-sensitizing DTX-resistant cells, enhancing the treatment's ability to prolong life in men with metastatic castration-resistant prostate cancer.

Preclinical evidence suggested that hydroxychloroquine (HCQ) and suba-ITZ (SI) could help men with biochemical relapses of prostate cancer avoid the side effects of androgen deprivation therapy (ADT). This phase I/II study looked at the safety, pharmacokinetics (PK), maximum tolerated dosage (MTD), recommended phase II dose (RP2D), and preliminary activity of HCQ/SI in these patients. In a rolling six design, patients were given increasing dosages of HCQ with a fixed SI of 150 mg BD. A phase II Simon 2-stage cohort expansion is scheduled to follow. HCQ/SI was used to treat eleven men. The baseline PSA was 4.4 µg L^−1^ (1.6–22.4), the median age was 73 (range 69–77), and the doubling time was 5.3 months (3.3–15.3). At HCQ 600 mg BD, two patients had dose-limiting toxicity: grade 3 alanine transferase increase and grade 3 diarrhea. The most frequent adverse events (AEs) associated with medication were QTc prolongation (55%/0%), nausea (36%/0%), diarrhea (36%/9%), and hypertension (91% all grade/18% grade 3). No grade 4 adverse events or fatalities occurred. Despite the lack of PSA responses (≥50% decline from baseline), the PSA doubling time was extended at 4 and 12 weeks in 82% and 45% of cases, respectively, and the PSA PFS according to PCWG3 criteria was 5.5 months (2.0–9.0). The survival time without ADT is 14.3 months (95% CI 4.9–23.8), and the survival time without metastases is 15.9 months (95% CI unevaluable). We'll show the PK data. When combined with MTD 600 mg BD and RP2D 400 mg BD, HCQ/SI showed adequate safety.^86^

ITZ nanoparticles (ITR NP) were created in this study using quality by design and multivariate analysis. They were assessed for cellular uptake, suppression of cell proliferation, and the mechanism of prostate cancer (PCa) cell inhibition. ITR NP's safety was demonstrated by time and concentration-dependent hemolytic potential and serum stability. The effectiveness of ITR and ITR NP in inducing growth inhibition of PC-3 cells was demonstrated by morphological changes and nuclear staining investigations. Compared to the ITR and control groups, ITR NP showed superior qualitative and quantitative absorption, ROS, and mitochondrial damage. A cell cycle analysis showed that PC-3 cells exhibited a notable suppression of the G2/M phase. In 3D tumoroids that resembled micro-metastatic lesions, ITR NP showed better anticancer potential than both control and ITR. ITR NP may therefore be a good substitute treatment option for PCa.^87^

## Nasopharyngeal carcinoma cells, solid tumors, hemangioma endothelial cells, and tongue carcinoma

Xu, Ying, *et al.*^88^ collected the spheroids produced by NPC cells—which were shown to possess stem cell-like characteristics—they discovered that these spheroids showed some degree of radioresistance. Additionally, NPC spheroids showed some resistance to ferroptosis, as evidenced by an increase in glutathione (GSH) and a decrease in iron concentration in lipid peroxides and lysosomes. ITZ also caused ferroptosis in NPC spheroids, which is defined as an increase in iron concentration and lipid peroxide oxygen, a drop in GSH levels, and a reduction in NPC spheroids' cell viability. Notably, NPC spheroids' radioresistance was partially reversed by ITZ. In terms of mechanism, this study discovered that ITZ can cause ferroptosis by sequestering iron in the lysosome; this is necessary for ITZ-mediated attenuation of NPC spheroid stemness. Consequently, this work offers proof that it is possible to utilize ITZ to kill NPC stem cells and thereby reduce radioresistance.

This study assessed the interactions between the OATP1B/CYP3A inhibitor ritonavir or the potent CYP3A inhibitor ITZ and the antibody–drug combination trastuzumab deruxtecan (T-DXd; DS-8201a), which targets the human epidermal growth factor receptor 2 (HER2). Participants in this phase I, open-label, single-sequence crossover research (NCT03383692) with HER2-expressing advanced solid tumors were given intravenous T-DXd 5.4 mg kg^−1^ every three weeks. From day 17 of cycle 2 to the conclusion of cycle 3, patients were treated with either ITZ (cohort 2) or ritonavir (cohort 1). T-DXd + ITZ or ritonavir had a safety profile that was in line with earlier research on TDXd monotherapy. T-DXd showed encouraging anticancer efficacy against all solid tumor types that expressed HER2. ITZ or ritonavir was safely added to T-DXd without having a discernible effect on the pharmacokinetics of either drug.^89^

In this work, human umbilical vein endothelial cells were used as the control group to investigate how ITZ affected the proliferation, apoptosis, and angiogenesis of hemangioma endothelial cells (HemECs). Using a real-time quantitative polymerase chain reaction, the expression of the genes SHH, PTCH1, SMO, and GLI1 implicated in the hedgehog (HH) signaling pathway was ascertained. The expression of associated proteins was confirmed by western blotting. ITZ dramatically reduced the viability of HemECs in this investigation in a dose- and time-dependent manner. Inhibiting cell proliferation and angiogenesis, ITZ decreased the expression of PCNA, Ki67, and VEGF. Furthermore, ITZ caused HemECs to undergo apoptosis by upregulating BAX expression and suppressing BCL2 expression. The expression of SHH, PTCH1, SMO, and GLI1 was suppressed by ITZ. ITZ's effect on HemECs was lessened when the rhSHH protein activated the HH pathway. In summary, ITZ suppresses the HH signaling system, which in turn causes apoptosis, slows proliferation, and decreases angiogenesis of HemECs. As a result, ITZ might be a different option for treating infantile hemangioma (IH).^90^

The objective of this work was to assess the impact of ITZ on invasion and migration of TSCC cell line and to connect tumor grade and lymph node involvement with immunohistochemistry expression of α-SMA, a marker for cancer-associated fibroblasts (CAF) and TGF-β. Twenty-four samples with varying stages of TSCC and clinical lymph node involvement had their immunohistochemical expression of TGF-β and α-SMA assessed retrospectively. ITZ was molecularly docked with PI3/AKT, and its impact on SCC-25 cell lines grown in media derived from the co-cultivation of SCC-25 and WI-38 (normal fibroblast) cell lines was evaluated. TGF-β and α-SMA immunohistochemical expression was noticeably higher in those with lymph node involvement. ITZ and PI3/AKT proteins had a significant interaction. In comparison to the control group, the ITZ-treated group exhibited noticeably reduced α-SMA, TGF-β, SNAIL, and VEGF expressions, as well as migration and invasion capacity. *In vitro*, ITZ was able to prevent the TSCC cell line from invading and migrating.^91^

## Synergistic, additive and antagonistic effects of itraconazole with anticancer drugs

ITZ, participating in various signaling circuits such as antiangiogenic effects (VEGFR and mTOR pathways) and hedgehog signaling pathway inhibition, has demonstrated various effects on the efficacy of anticancer drugs and has been considered as an adjuvant in cancer therapy. Its synergistic effects with paclitaxel has been *via* these mechanisms against ovarian cancer cells.^92^ Additionally, its effects on P-gp mediated 99mTc-MIBI efflux pump has been associated with synergistic effect with paclitaxel against HT-29 colon cancer cells in nude mice with 73.3% of growth suppression compared to 60.02% for paclitaxel singly.^70^ Its synergistic effects with cisplatin has been also associated with related mechanisms in xenograft models of human lung cancer cells.^93^ Regarding hedgehog signaling pathway, its synergy with docetaxel has been inferred against prostate and MCF-7 drug-resistant-breast cancer cells, though prior mechanisms has been also stated.^85,94,95^ These effects have resulted in resistance reversal to overcome chemotherapy resistance. ITZ has also re-sensitized resistance to osimertinib by lung cancer cells *via* SHH/DUSP13B/p-STAT3 axis inhibition.^57^ Co-administration of ITZ and osimertinib had a negligible increase in *C*_max_ and AUC_0–*t*_ of osimertinib, while fluconazole and voriconazole had considerable increasing effects in these pharmacokinetics properties.^96^ However, ITZ has unveiled increased neurotoxicity in combination with vincristine offering implications in caution in their consumption.^97–100^ In addition, ITZ and its metabolites inhibit P450 enzymes which may affect the efficacy of anticancer drugs and exert cytotoxicity.^101,102^ Accordingly, ITZ has deciphered acceptable synergistic effects with various anticancer agents, including paclitaxel, cisplatin, and docetaxel, mainly through mechanisms such as antiangiogenic pathways, hedgehog signaling inhibition, and effects on drug efflux pumps. These interactions can increase chemotherapy efficacy and contribute to overcome resistance in diverse cancer types. Moreover, its ability to reverse resistance of lung cancer cells to osimertinib highlights its potential as an adjuvant therapy. However, caution is warranted due to its impact on P450 enzymes, which may alter drug pharmacokinetics, and the risk of increased neurotoxicity when combined with agents such as vincristine.

## Novel delivery approaches

ITZ faces considerable delivery challenges for its poor aqueous solubility and variable oral bioavailability. Its absorption is highly dependent on gastric pH and food intake, resulting in inconsistent plasma levels.^103^ The drug's metabolism by the liver enzyme CYP3A4 also leads to drug–drug interactions.^104^ Additionally, ITZ has limited penetration into some types of tissues, restricting its effectiveness.^105^ Moreover, formulation difficulties are of its hydrophobic nature, complicating intravenous and oral delivery. These limitations often necessitate dose adjustments and cautious monitoring to ensure therapeutic efficacy while minimizing toxicity. However, there are various drug delivery approaches such as cyclodextrin complexes (increasing stability and solubility), pH-sensitive formulations (for oral delivery), targeted delivery systems, intravenous formulations and lipid-based formulations to increase ITZ efficacy.^106–108^

## Comparative critique of itraconazole *versus* other azoles and standard chemotherapies

Among repurposed azole antifungals, ITZ is unique because of its multi-targeted anticancer actions and comparatively advanced translational evidence. ITZ modulates a number of cancer-relevant pathways, such as hedgehog signaling, angiogenesis, cholesterol trafficking, autophagy, and mTOR-related processes, in contrast to ketoconazole, whose anticancer application is restricted by hepatotoxicity and endocrine side effects, or fluconazole and voriconazole, which show weak and inconsistent anticancer activity. Posaconazole's anticancer proof is still mostly preclinical, despite the fact that it shares certain molecular similarities with ITZ.

ITZ has a better safety profile, oral administration, and cost-effectiveness when compared to conventional chemotherapeutic drugs. Its effectiveness as a monotherapy, however, is typically limited, indicating that it would be better used as an adjuvant or combination treatment rather than as a substitute for cytotoxic chemotherapy. CYP3A4 inhibition-mediated drug–drug interactions continue to be a significant restriction that calls for cautious therapeutic assessment (Table 2).

**Table 2:** Comparative overview of itraconazole, other repurposed azoles, and standard chemotherapies

Aspect	Itraconazole (ITZ)	Other repurposed azoles	Standard chemotherapies	Ref.
Primary anticancer mechanisms	Hedgehog inhibition, anti-angiogenesis, cholesterol trafficking disruption, autophagy/mTOR modulation	Ketoconazole: CYP450 & androgen inhibition; fluconazole/voriconazole: weak or indirect effects	DNA damage, mitotic inhibition, apoptosis induction	9 and 18
Breadth of pathway targeting	High (multi-targeted)	Low to moderate	Typically, a single dominant cytotoxic mechanism	9 and 109
Level of cancer-related evidence	Preclinical and early clinical trials	Mostly preclinical, limited translational data	Extensive clinical and regulatory validation	9 and 110
Toxicity profile	Generally well-tolerated; CYP3A4 interactions	Ketoconazole: hepatotoxicity; others variable	High systemic toxicity, narrow therapeutic window	19 and 109
Therapeutic role	Adjuvant or combination therapy	Experimental	First-line or standard of care	110
Cost and accessibility	Low cost, orally available	Moderate	Often high cost	109

## Conclusion

Cancer is a growing problem in terms of deaths and, to a lesser extent, costs, as evidenced by a thorough analysis of statistics detailing diagnosis, mortality, and financial expenditures in the field of oncology. Neoplasms' capacity for invasion and metastasis is what causes patients' pain and subpar clinical results. As a novel anti-tumor treatment, ITZ is a well-known antifungal drug that has been thoroughly investigated for its capacity to inhibit various signaling pathways such as Hh. Numerous malignancies have been effectively treated with this medication with promising outcomes. In addition, it inhibits Hh signaling and seems to have several molecular targets, which lowers resistance in contrast to drugs that only target one target. ITZ's antitumor effects have been proven by evidence from *in vitro*, *in vivo*, and clinical trials, which have also identified several important pathways as potential targets. It has been documented that it induces apoptosis or autophagy in glioblastoma and esophageal, gastric, colon, pancreatic, breast, and epithelial ovarian malignancies. These findings enable ITZ to boost medication efficacy and combat drug resistance, either by itself or in conjunction with other chemotherapeutic drugs. ITZ must be used in conjunction with other medications that impact cell survival, even though trials are presently being conducted and further research is anticipated. In tumors linked to drug resistance and other cancers known to be impacted by the hedgehog pathway and angiogenesis, they must use ITZ for extended periods of time and at different phases of the disease.

## Future perspectives and outlook

ITZ is a promising repurposed anti-cancer medication, but before it can reach its full therapeutic potential, a number of important issues and possibilities need to be resolved. In order to maximize anticancer efficacy while reducing off-target effects and drug–drug interactions, future research should focus on developing a deeper knowledge of the structure–activity relationships (SAR) associated with ITZ and its derivatives. New analogues with improved selectivity for cancer-related signaling pathways could be created by rationally altering its chemical structure.

Although ITZ has been demonstrated to influence several carcinogenic pathways at the mechanistic level, such as hedgehog, Wnt/β-catenin, angiogenesis-related signaling, and autophagy, its specific molecular targets in various cancer types are still unclear. It is anticipated that advanced omics techniques, systems biology, and target deconvolution techniques would be essential in clarifying context-dependent mechanisms of action and locating response-predictive biomarkers.

From a therapeutic perspective, future clinical research should concentrate on well planned, cancer-specific trials to identify the best dosage schedules, length of therapy, and combination approaches. Specifically, ITZ in combination with immunotherapies, targeted treatments, or chemotherapy is a viable way to improve therapeutic outcomes and overcome resistance. ITZ 's therapeutic applicability may also be increased by enhancing its pharmacokinetic profile and bioavailability using cutting-edge drug delivery methods like prodrug techniques or nanocarriers.

Finally, the successful transition of ITZ from a repurposing candidate to mainstream oncological therapy will depend on cost-effectiveness assessments, real-world evidence, and regulatory concerns. Ultimately, realizing ITZ 's full potential as a flexible and reasonably priced anti-cancer therapy in the future would need combining chemical optimization, molecular insights, and solid clinical confirmation.

## Author contributions

Mahrokh Marzi, and Elham Zarenezhad designed and supervision this study. The first draft of the manuscript was written by Mahrokh Marzi, Abdolmajid Ghasemian, Ali Ghanbariasad, Ensieh Nournia, and Elham Zarenezhad. All authors approved the final manuscript.

## Conflicts of interest

The authors declare that they have no competing interests.

## Abbreviations

ABC: ATP-binding cassette
ADMET: Absorption, distribution, metabolism, excretion and toxicity
ADT: Androgen deprivation therapy
AEs: Adverse events
ALP: Alkaline phosphatase
ALT: Alanine aminotransferase
AMPK: 5′ AMP-dependent protein kinase
AP: Ascorbyl palmitate
ARB: Angiotensin receptor blocker
BALF: Bronchoalveolar lavage fluid
BCC: Basal cell carcinoma
BE: Barrett's esophagus
BM: Barrett's metaplasia
CAF: Cancer associated fibroblast
CRC: Advanced colorectal cancer
cSCC: Cutaneous squamous cell carcinoma
CYP: Cytochrome P450 monooxygenase
ECOG: Eastern Cooperative Oncology Group
ENO1: Enolase 1
EOC: Epithelial ovarian cancer
EPR: Enhanced permeability and retention
FDA: Food and Drug Administration
GLI: Glioma-associated oncogene homologs
GLY: Glycerosomes
HA: Hyaluronic acid
HCQ: Hydroxychloroquine
HemECs: Hemangioma endothelial cells
Hh pathway: Hedgehog pathway
HMGCS1: 3-Hydroxy-3-methylglutaryl-CoA synthase 1
IH: Infantile hemangioma
ITZ: Itraconazole
ITR NP: Itraconazole nanoparticles
LC/MS: Liquid chromatography/mass spectrometry
LNC: Lipid nanocapsules
MDR: Multidrug-resistant
mPC: Metastatic pancreatic cancer
MTD: Maximum tolerated dosage
mTOR: Mechanistic target of rapamycin
NIM: Nanoparticles-in-microparticles
NHF: Normal human fibroblasts
NSCLC: Non-small cell lung cancer
NPC: Niemann–Pick type C
ORR: Overall response rate
OS: Overall survival
PCa: Prostate cancer
PDAC: Pancreatic ductal adenocarcinoma
PFS: Progression-free survival
P-gp: P-glycoprotein
PK: Pharmacokinetics
PMF: Probabilistic matrix factorization
PVA: Polyvinyl alcohol 500
QbD: Quality by design
rhShh: Recombinant human Sonic hedgehog
ROS: Reactive oxygen species
RP2D: Recommended phase II dose
SCP2: Sterol carrier protein 2
SD: Sprague-Dawley
SHH: Sonic hedgehog
SMO: Smoothened receptors
SPMs: Specialized pro-resolving mediators
SUFU: Suppressor of fused
TAMs: Tumor-associated macrophages
T-DXd: Trastuzumab deruxtecan
TNBC: Triple negative breast cancer
TNF: Tumor necrosis factor
VDAC1: Voltage-dependent anion channel 1
VEGF: Vascular endothelial growth factor
VEGFR2: Vascular endothelial growth factor receptor 2

## Data Availability

No primary research results, software or code have been included and no new data were generated or analysed as part of this review.
